# Mobile Apps for Weight Management: A Scoping Review

**DOI:** 10.2196/mhealth.5115

**Published:** 2016-07-26

**Authors:** Jordan Rivera, Amy McPherson, Jill Hamilton, Catherine Birken, Michael Coons, Sindoora Iyer, Arnav Agarwal, Chitra Lalloo, Jennifer Stinson

**Affiliations:** ^1^ Child Health Evaluative Sciences The Hospital for Sick Children Toronto, ON Canada; ^2^ Lawrence S. Bloomberg Faculty of Nursing University of Toronto Toronto, ON Canada; ^3^ Bloorview Research Institute Holland Bloorview Kids Rehabilitation Hospital Toronto, ON Canada; ^4^ Dalla Lana School of Public Health University of Toronto Toronto, ON Canada; ^5^ Physiology and Experimental Medicine The Hospital for Sick Children Toronto, ON Canada; ^6^ Department of Paediatrics Faculty of Medicine University of Toronto Toronto, ON Canada; ^7^ Department of Family and Community Medicine Faculty of Medicine University of Toronto Toronto, ON Canada

**Keywords:** weight loss, obesity, mobile apps, smartphones, mHealth

## Abstract

**Background:**

Obesity remains a major public health concern. Mobile apps for weight loss/management are found to be effective for improving health outcomes in adults and adolescents, and are pursued as a cost-effective and scalable intervention for combating overweight and obesity. In recent years, the commercial market for ‘weight loss apps’ has expanded at rapid pace, yet little is known regarding the evidence-based quality of these tools for weight control.

**Objective:**

To characterize the inclusion of evidence-based strategies, health care expert involvement, and scientific evaluation of commercial mobile apps for weight loss/management.

**Methods:**

An electronic search was conducted between July 2014 and July 2015 of the official app stores for four major mobile operating systems. Three raters independently identified apps with a stated goal of weight loss/management, as well as weight loss/management apps targeted to pediatric users. All discrepancies regarding selection were resolved through discussion with a fourth rater. Metadata from all included apps were abstracted into a standard assessment criteria form and the evidence-based strategies, health care expert involvement, and scientific evaluation of included apps was assessed. Evidence-based strategies included: self-monitoring, goal-setting, physical activity support, healthy eating support, weight and/or health assessment, personalized feedback, motivational strategies, and social support.

**Results:**

A total of 393 apps were included in this review. Self-monitoring was most common (139/393, 35.3%), followed by physical activity support (108/393, 27.5%), weight assessment (100/393, 25.4%), healthy eating support (91/393, 23.2%), goal-setting (84/393, 21.4%), motivational strategies (28/393, 7.1%), social support (21/393, 5.3%), and personalized feedback (7/393, 1.8%). Of apps, 0.8% (3/393) underwent scientific evaluation and 0.3% (1/393) reported health care expert involvement. No apps were comprehensive in the assessment criteria, with the majority of apps meeting less than two criteria.

**Conclusions:**

Commercial mobile apps for weight loss/management lack important evidence-based features, do not involve health care experts in their development process, and have not undergone rigorous scientific testing. This calls into question the validity of apps’ claims regarding their effectiveness and safety, at a time when the availability and growth in adoption of these tools is rapidly increasing. Collaborative efforts between developers, researchers, clinicians, and patients are needed to develop and test high-quality, evidence-based mobile apps for weight loss/management before they are widely disseminated in commercial markets.

## Introduction

### Background

The prevalence of overweight and obesity worldwide is predicted to exceed 1.12 billion and 573 million people, respectively, by 2030 [1]. Excess weight is closely linked to a myriad of chronic diseases such as hypertension, type 2 diabetes mellitus, cardiovascular disease, and stroke [2]. The economic costs of obesity globally are estimated at 0.7% to 2.8% of total health care expenditures [3] and the burden of mortality is estimated at 2.8 million deaths annually [4]. Environmental changes promoting intake of highly caloric, inexpensive, nutrient dense foods, and larger portion sizes, coupled with decreased physical activity and increased sedentary behaviors are significant causative factors for obesity [5]. Accordingly, efforts to curb obesity have aimed to promote adherence to evidence-based recommendations for daily exercise, healthy eating, and associated behavioral determinants of weight [6].

Clinical interventions for obesity have demonstrated variable efficacy, which has been primarily attributed to fluctuations in treatment adherence over time [7,8]. Clinical interventions employ evidence-based strategies primarily based on behavior change theory to drive permanent lifestyle modifications necessary for long-term weight control [9]. These strategies include self-monitoring, goal-setting, healthy eating training, increasing physical activity, providing personalized and objective feedback, stress reduction, and problem solving [10]. Clinical interventions for obesity are rigorous and typically require face-to-face contact often for over a year [11]. These programs can be time, cost, and resource intensive, and often inconvenient for patients to attend, limiting long-term treatment adherence and weight maintenance [10,11]. Novel, low-cost, and widely accessible tools are needed to support the practice of evidence-based strategies for weight control [12], particularly when patients face significant barriers to accessing clinical treatments.

To address these concerns, efforts have shifted to emerging interactive information and communication technologies as a novel means to support chronic disease self-management with the potential for low-cost scalability [10-13]. Clinical intervention strategies can be translated to mobile devices, such as mobile apps, leveraging the multifunctional capabilities and widespread use of mobile devices [10,14]. Growing research supports the ability for mobile devices to deliver effective intervention strategies for weight loss [13,15-17]. Mobile devices purposed toward improvements in weight, diet, and physical activity have demonstrated superior effectiveness on weight outcomes and behavioral determinants of weight when compared with standard no-intervention controls as well as to controls receiving nonmobile device interventions [17]. Mobile devices can be used to enhance self-efficacy by priming behavior activation and reducing the burden of behavior change techniques, such as providing convenient ways to self-monitor, set and update goals, communicate with supports, and access personally relevant education and resources efficiently [17].

Health researchers have started to develop and test their own mobile apps for weight management, with the objective to create new clinical and research tools that incorporate evidence-based strategies used in the treatment of obesity. A review of behavior change techniques in 12 primary trials and five secondary analyses examining mobile device interventions for weight loss found that interventions contained a minimum of five techniques, the most common of which were self-monitoring, goal-setting, tailored feedback, general health information, encouragement, prompting practice, and social support [17]. Mobile device interventions with multiple techniques that differentiated it meaningfully from the comparison treatment were associated with superior weight and health behavior outcomes [17].

However, studies that have explored commercial app markets have found that mobile apps for weight loss typically incorporate only a minority of the evidence-based strategies used in the treatment of obesity [11,18-21]. It is likely that the lack of evidence-based features limits the effectiveness of these tools, which is concerning given the abundance and availability of such tools for assisting with the general public’s weight management needs.

### Objective

To our knowledge, no studies have systematically and comprehensively explored the current commercial mobile app market to examine evidence-based strategies, health care expert involvement, and scientific evaluation of weight loss/management apps since these earlier investigations. The rapid growth in the number of health and fitness apps, combined with an increase in adoption of these tools in recent years underscores the need for an updated assessment of this rapidly changing market. Prior studies that have examined the inclusion of evidence-based strategies in commercial mobile apps for weight loss have mostly confined their search to a limited sample of apps (eg, <50) [11,18], to a single app store (eg, iTunes) [18-21], to a single population (eg, children) [20,21], or require updating (eg, published more than 3 years ago) [19]. The objective of the current study was to conduct an updated and comprehensive systematic review of weight management mobile apps across four major commercial app stores to describe the inclusion of evidence-based strategies for weight control, health care expert involvement, and scientific evaluation. Findings from this study will be used to identify the major overarching strategies relied upon by current weight management apps in order to provide direction for the advancement of research and app development.

## Methods

### App Search

**Figure 1:**
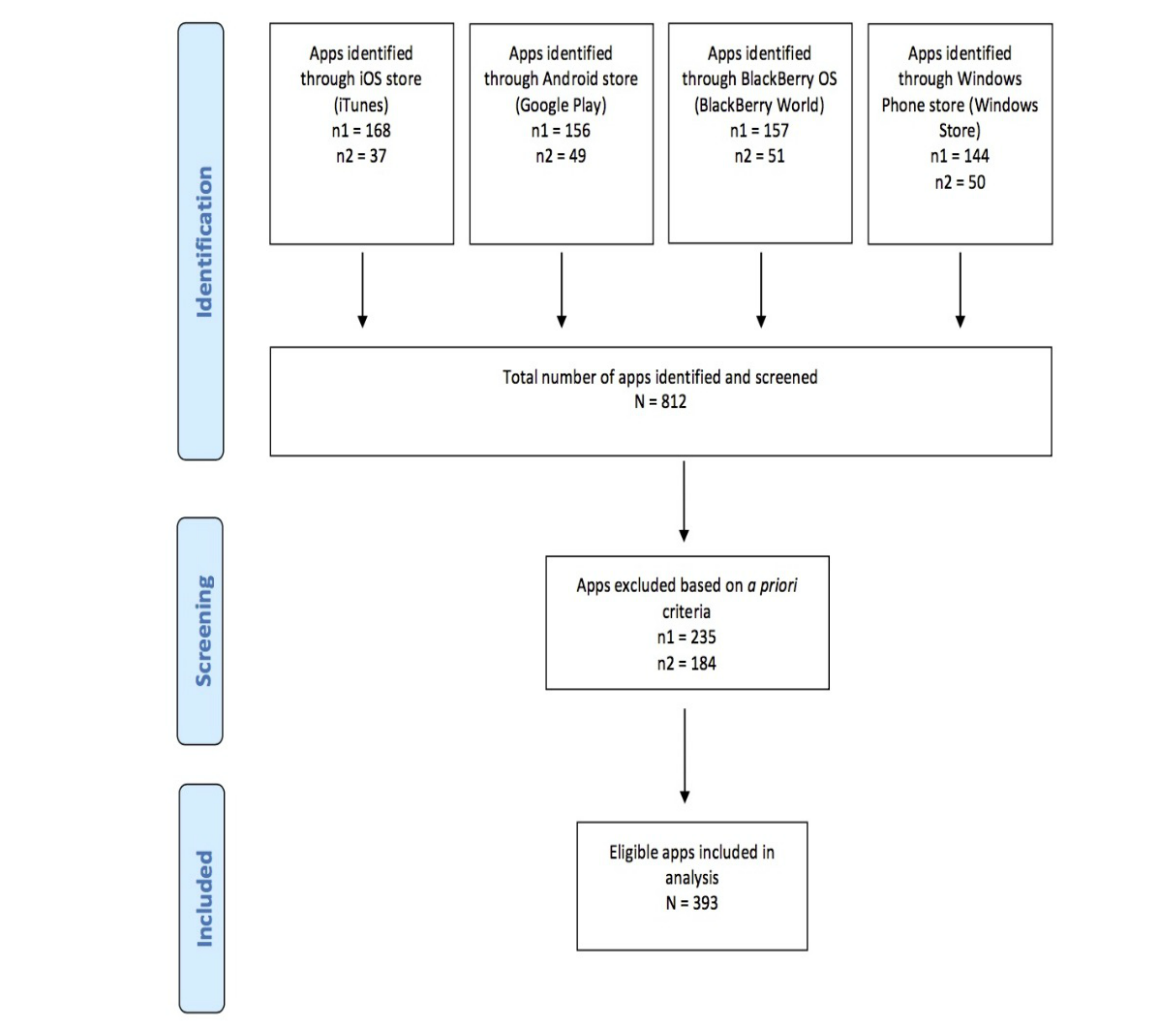
Flow diagram of study methods.

Figure 1 displays the methodology used in this study, which replicates that used in studies evaluating the functionality and content of mobile apps for health management [11,18-23]. An electronic search was conducted between July 2014 and July 2015 of the official app stores for iPhone operating systems (OS; iTunes), Android (Google Play), BlackBerry OS (BlackBerry World), and Windows Phone (Windows Store). Stores were searched separately using the search term ‘weight loss’ and no restrictions related to store subcategories were imposed. A secondary search was also performed to identify apps intended for children and adolescents using the search term ‘weight loss kids’. Results of the searches were not limited by language and no date of app publication was used to restrict search results. Because the indexing of apps varied greatly across app stores, prior to app selection we performed a calibration exercise in order to validate our ability to detect weight management apps. This comprised testing our search term for the ability to produce app results that were known to meet the inclusion criteria and be currently available in the app store for download (ie, if a search of ‘weight loss’ produced results that included a popular, widely known weight loss app such as Lose It!). In all cases, our search term identified the apps we expected to find.

### Selection of Apps

Apps met study inclusion criteria if the purpose of the app involved weight loss or weight management and the primary intended app user was a person seeking to reduce or manage their weight. Apps appearing in more than 1 app store were rated independently in order to account for differences in features supported by different mobile OS. For our secondary search, the app also needed to state its intended user to be a child, adolescent, teenager, young person, or youth. Apps were excluded if they were classified as “e-books” by the respective app store or were judged by the reviewers as such. Three investigators performed app selection independently and all discrepancies regarding selection were resolved through discussion with a fourth party. There was greater than 95% agreement between authors across all app stores prior to fourth author resolution.

### Data Abstraction

Metadata from all included apps were abstracted into a standard Microsoft Excel

spreadsheet. Metadata were collected from the respective app store where the app was identified. Abstracted metadata included: app name, developer (individual or organization), and price. A systematic approach to data abstraction was used. Specifically, two investigators independently collected data from each app store and each dataset was then cross-verified against the other.

### Evidence-Based Strategies

Apps’ evidence-based strategies were characterized based on inclusion/exclusion of app features or informational content selected through consensus by our team of obesity experts. The set of features/content used to characterize apps’ evidence-based strategies are supported by public health recommendations [19-21], widely disseminated clinical interventions [11,24], research in behavior change theory [9,17,18,25], as well as systematic reviews and meta-analyses of mobile device intervention studies [12,13,15-17,26-32]. While this particular list of features does not address every evidence-based strategy used in the management of obesity, our goal was to create broad categories representing the major aspects of evidence-based treatment in order to describe the overarching evidence-based quality of the current market for weight loss/management apps.

The following eight evidence-based strategies were examined: presence of (1) self-monitoring capabilities [11,12,15, 17-19,25,26,28] for weight, meals, nutrition (including protein, fats, carbohydrates, fiber, and water), physical activity, cardiometabolic indicators, sleep, mental health indicators, including mood, thought patterns, cognitions, and stress, environmental influences, and custom metrics, (2) goal-setting [11,15,17,18,25] with/without customization, (3) healthy eating support [11,15,17-19,25,29], including information, education, and skills development, (4) physical activity support [11,15,17-19,25,29], including information, education, and skills development, (5) social support [12,15,17-19,26,30], (6) weight and/or health assessment [11,17,19], with/without personalization, (7) motivational strategies [15,17,18], including prompts, rewards, or gamified design, and (8) personalized feedback [12,15,17,26,30].

### Health Care Expert Involvement and Scientific Evaluation

Health care expert involvement in app development and stated involvement in formal scientific evaluation were also examined. An app was required to reference the involvement of a health care professional listed in the Ontario Regulated Health Professions Act [33] and app descriptions as well as publicly accessible scientific literature databases (ie, National Center for Biotechnology Information PubMed and Google Scholar) were searched by app name for any published research related to the app. For each category, we assessed inclusion as either ‘present’ or ‘absent’. Descriptive statistics were used to summarize the results of the assessment.

## Results

### Summary of Search Results

Our first search across all app stores identified a total of 625 apps. Of these apps, 37.6% (235/625) were excluded from further review based on the a priori inclusion and exclusion criteria. The primary reason for app exclusion was the inability to provide any weight management support (ie, the app was used exclusively as a game). A total of 390 apps were included in the primary analysis. Our secondary search identified a total of 187 apps. Of these apps, 184 were excluded based on the a priori inclusion and exclusion criteria, which also required the app’s intended user to be a child, adolescent, teenager, young person, or youth. A total of three apps were included in the secondary analysis. In total, 393 apps were included in the final analysis.

### Summary of General App Characteristics

Overall, identified apps were most often classified as ‘medical’, ‘lifestyle’, or ‘health and fitness’. The cost of apps also varied, with approximately 87.3% (343/393) being free to download. The cost of paid apps ranged from $0.99 to $7.99 CDN.

### Assessment of Evidence-Based Strategies

Table 1 shows the frequency of evidence-based strategies across included apps. The most common strategy was self-monitoring (139/393, 35.3%), which allowed the user to track targeted weight-related metrics over time, the majority of which consisted of weight, energy balance, water intake, and quantity of physical activity. Few apps included more comprehensive tracking options such as nutrition, sleep, and cardiometabolic indicators. No apps allowed for tracking of mental health indicators, environmental influences, or allowed for the creation of customized metrics. Within these apps, 10.2% (40/393) could automatically monitor the user’s physical activity without the requirement for manual logging. The second most common strategy was physical activity support (108/393, 27.5%), which mostly included fitness plans, exercise guides, and tracking of daily physical activity. Of apps, 25.4% (100/393) included weight and/or health assessment, which was limited to assessment of body mass index (BMI). No other types of health assessment capabilities were found. Of apps, 23.2% (91/393) provided healthy eating support, most commonly healthy eating guidelines, meal plans, calorie balance goals, and nutritional information for specific foods. No apps included skills development needed for healthy eating such as stress reduction, emotion regulation, stimulus control, time management, and problem solving. Of apps, 21.4% (84/393) included goal-setting, which mainly consisted of weight loss goals, calorie balance goals, water intake goals, and physical activity goals. No apps allowed for the creation of customized goals. Of apps, 7.1% (28/393) possessed motivational strategies including prompts, gamification, or use of rewards (ie, points for meeting weight goals). Of apps, 5.3% (21/393) featured a social support component such as online communication with other users. Lastly, 1.8% (7/393) of apps provided personalized feedback to the user, such as through virtual meetings with a health coach or through notifications.

### Health Care Expert Involvement and Scientific Evaluation

As shown in Table 1, only 0.3% of apps (1/393), *Kurbo Health,* stated the involvement of a regulated health care professional in the app’s development. This app reported involving a medical advisory board consisting of pediatricians, psychologists, and psychiatrists. Furthermore, only 0.8% of apps (3/393) were found to have been part of formal scientific research or have undergone scientific testing.

### Pediatric Focused Weight Management Apps

Our search identified only three pediatric focused apps. Two of the apps lacked the majority of evidence-based strategies for weight management. *Ideal Weight BMI Adult and Child* only provided assessment of BMI and *Choose My Food* used only a gamified design. The third app, *Kurbo Health,* possessed 8 strategies including self-monitoring, goal-setting, physical activity support, healthy eating support, social support, gamification, and personalized feedback delivered via a health coach (offered as a premium paid-for feature).

**Table 1:** Evidence-based strategies, health care expert involvement, and scientific testing in apps for weight management.

Criteria	iTunes (n=95)	Google Play (n=98)	Windows Store (n=100)	Blackberry World (n=100)	Total (N=393)
	n (%)	n (%)	n (%)	n (%)	N (%)
Self-monitoring	47 (49.5%)	40 (40.8%)	28 (28.0%)	24 (24.0%)	139 (35.4%)
Automatic self-monitoring	9 (9.5%)	2 (2.0%)	13 (13.0%)	16 (16.0%)	40 (10.2%)
Goal-setting	29 (30.5%)	30 (30.6%)	14 (14.0%)	11 (11.0%)	84 (21.4%)
Physical activity support	26 (27.4%)	30 (30.6%)	25 (25.0%)	27 (27.0%)	108 (27.5%)
Healthy eating support	16 (16.8%)	47 (48.0%)	7 (7.0%)	21 (21.0%)	91 (23.2%)
Weight /health assessment	21 (22.1%)	24 (24.5%)	24 (24.0%)	31 (31.0%)	100 (25.4%)
Personalized feedback	1 (1.1%)	2 (2.0%)	4 (4.0%)	0 (0.0%)	7 (1.9%)
Motivational strategies (rewards, prompts, or gamification)	9 (9.5%)	7 (7.1%)	11 (11.0%)	1 (1.0%)	28 (7.1%)
Social support	4 (4.0%)	7 (7.1%)	10 (10.0%)	0 (0.0%)	21 (5.3%)
Health care expert involvement	1 (1.2%)	0 (0.0%)	0 (0.0%)	0 (0.0%)	1 (0.3%)
Scientific testing	0 (0.0%)	1 (1.0%)	2 (2.0%)	0 (0.0%)	3 (0.8%)

### Comprehensiveness of Weight Management Apps for Assessment Criteria

As shown in Table 2, the relative representation of assessment criteria including evidence-based strategies, health care expert involvement, and scientific evaluation per app varied. Roughly one-third of apps (130/393, 33.1%) did not meet any of the assessment criteria. Just over one-quarter of apps (103/393, 26.2%) met one criterion. The remaining apps are described as follows: 16.8% (66/393) met 2 criteria, 14.8% (58/393) met 3 criteria, 5.1% (20/393) met 4 criteria, 2.3% (9/393) met 5 criteria, 0.8% (3/393) met 6 criteria, 0.5% (2/393) met 7 criteria, and 0.5% (2/393) met 8 criteria. No app met more than 8 of our assessment criteria. The average number of criteria present in an app was between 1 and 2. In general, most apps functioned as either a fitness app or a dieting app.

**Table 2:** Comprehensiveness of apps for assessment criteria.

# of criteria met	iTunes (n=95)	Google Play (n=98)	Blackberry World (n=100)	Windows Store (n=100)	Total (N=393)
	n (%)	n (%)	n (%)	n (%)	N (%)
0	39 (41.1%)	9 (9.2%)	38 (38.0%)	44 (44.0%)	130 (33.1%)
1	13 (13.7%)	41 (41.8%)	25 (25.0%)	24 (24.0%)	103 (26.2%)
2	9 (9.5%)	23 (23.5%)	22 (22.0%)	12 (12.0%)	66 (16.8%)
3	23 (24.2%)	11 (11.2%)	14 (14.0%)	10 (10.0%)	58 (14.8%)
4	6 (6.3%)	6 (6.1%)	1 (1.0%)	7 (7.0%)	20 (5.1%)
5	2 (2.1%)	6 (6.1%)	0 (0.0%)	1 (1.0%)	9 (2.3%)
6	2 (2.1%)	0 (0.0%)	0 (0.0%)	1 (1.0%)	3 (0.8%)
7	0 (0.0%)	2 (2.0%)	0 (0.0%)	0 (0.0%)	2 (0.5%)
8	1 (1.1%)	0 (0.0%)	0 (0.0%)	1 (1.0%)	2 (0.5%)
9	0 (0.0%)	0 (0.0%)	0 (0.0%)	0 (0.0%)	0 (0.0%)
10	0 (0.0%)	0 (0.0%)	0 (0.0%)	0 (0.0%)	0 (0.0%)

## Discussion

### Principal Findings

This review demonstrates that despite the abundance of mobile apps for weight management, the evidence-based quality of these apps remains generally poor. The rapid growth in the commercial market for weight management apps has outpaced progress to improve the content and functionality of these tools, creating an overabundance of weight management apps with no evidence base being made readily available to the public.

We can summarize the major limitations of current user-focused apps for weight management, characterized by: (1) simplistic capabilities that lack high-level personalization to complex user needs and preferences, (2) a lack of health care expert involvement during app development, (3) minimal use of evidence-based strategies for the management of obesity, and (4) the absence of scientific evaluation of these tools.

Current capabilities for promoting behavior change for weight management through mobile apps have low fidelity and do not reflect the individually tailored practices employed in clinical obesity interventions. Apps tend to possess a singular focus on either the physical activity or dieting practices for weight loss. Moreover, apps do not comprehensively address the full range of cognitive, behavioral, and environmental factors that can impact a person’s ability to manage their weight over the long term. These findings are reflected in similar work by numerous authors [11,18-21] who also report a low level of adherence to evidence-based practices in mobile apps for weight loss and the lack of cognitive and behavioral targets related to weight control. This suggests that limited improvements have been made in the commercial app market since these studies, despite the growth in the number of apps available. The lack of evidence-based strategies employed by the apps we reviewed drastically narrows the therapeutic scope of these tool for addressing the multifactorial causes of overweight and obesity. Hence, most apps may not be suitable for supporting individuals with severe or complex obesity who have complex medical and self-management needs. This may be in part due to the general lack of health care expert involvement during app development or that the majority of commercial apps are not developed with the intention for use by clinical populations. Only three apps, *My Fitness Pal*, *Lost It!*, and *Fitbit* were found to be involved in formal scientific research. The lack of rigorous scientific evaluation by most apps calls into question the validity of apps’ claims regarding their therapeutic benefit and safety, as well as encourages the rapid development and sale of medical apps with no demonstrated clinical efficacy.

Evidence-based strategies are critical to most clinical weight loss programs but were largely absent from the mobile apps we reviewed. Approximately one-third of the apps included self-monitoring and physical activity support, less than one-quarter facilitated goal-setting or provided healthy eating support, less than one-tenth contained motivating components or provided appropriate social support, and only 1.8% (7/393) provided personalized feedback to the user. While research has yet to be conducted into the appropriate and optimal number of strategies employed by a single app, effective long-term weight management requires a range of behavioral and lifestyle changes in order to address the multifactorial etiology of obesity.

Goal-setting and self-monitoring are key strategies derived from self-regulation theory to enhance self-efficacy and significantly predict weight loss and behavior change success [17,18]. Apps with goal setting focused mainly on weight, calorie, or exercise goals and generally could not be customized to personal objectives or personalized to user preferences. Most apps with self-monitoring features focused on tracking activity, meals, and calories, but important metrics related to nutrition, cardiovascular health, sleep, mental health, and environmental influences, as well as custom metrics, were mostly neglected. Moreover, the self-monitoring capabilities of current mobile apps are limited by the manual input demands on the user, requiring that users remember and be consistently motivated to input multiple types of data frequently in order to be successful. Providing personalized feedback to the individual on their progress as well as facilitating social support from peers are strategies routinely performed in intensive behavioral interventions to improve long-term success in goal-setting and self-monitoring [17]. These important features are mostly absent from current mobile apps, which do not possess the functional capabilities to deliver this complex level of interaction and communication. Furthermore, the limited amount of content and features dedicated to healthy eating is concerning given the pivotal role nutritional factors play in obesity management.

### Limitations

The search methods used in this review were modeled after those previously conducted in this area [11,18-21] and in the management of other chronic diseases [22]. Our search protocol was intended to mimic the search experience of a general user who would most likely follow a similar strategy when choosing apps for their own weight management. Although we attempted a comprehensive abstraction of data related to app content and functionality, reviewers did not download apps onto a smartphone device for thorough review. Rather, information was gathered from the app store and from associated websites. Hence, the data presented should not be interpreted to reflect the accuracy of any particular feature (ie, accuracy of energy expenditure measurement), as this was not the aim of the present study. These findings reflect the general knowledge of app developers regarding practices for effective weight management, as well as the evidence-based quality of current commercial weight loss apps. These findings provide an overview of the current state of the mobile weight loss app market in order to guide the future direction of research and development of these tools. Moreover, the findings reported are very broad characterizations of current app features meant for weight management, yet the specific strategies relied upon, such as specific nutritional content offered by the app, is not described. Future research will be required to determine the accuracy of particular app features and to characterize the specific types of strategies employed within each of the reported criteria categories.

### Future Directions

Our findings contribute to the growing work into the development and evaluation of mobile- and Internet-based tools for overweight and obesity management. The challenges to sustaining clinic-based interventions in the long-term, in addition to the near-ubiquity and multifunctional capabilities of today’s smartphone devices position mobile apps as a potential translational platform for the widespread delivery of weight management interventions. Numerous studies now support the ability for mobile phones to facilitate weight loss and promote associated healthy behaviors [12,13,15-17,26-31], as well as reduce obesity-related comorbidities [32]. While these benefits have been observed in clinical trials involving researcher-developed apps, limited data exist evaluating the quality of commercial mobile apps and their inclusion of evidence-based strategies for weight loss/management. Our findings demonstrate that while significant progress has been made in the development and accessibility of mobile apps for weight loss, considerable improvements are needed before these tools can truly be considered evidence-based.

Future efforts by both researchers and commercial developers should aim to address the limitations discussed. More stringent standards for the provision of medical apps should be established and incorporated into the process of submission to an app store. More comprehensive use of evidence-based strategies used in routine behavioral counselling for weight loss should be integrated into apps’ functionality and content. This is not a straightforward objective because many of these strategies would require complex, intelligent interaction with the device (eg, such as providing tailored feedback) and would also need to be adapted to the usability constraints of a mobile device interface (eg, screen size). However, the potential outcome of these efforts would be that mHealth interventions would become personalized to the user’s needs for managing their health (eg, tailored to the patient’s lifestyle), rather than providing generic and homogenous support to every user. In addition, health care experts need to become more integral to the development and distribution of medical apps. The concerns of any medical treatment, such as safety and efficacy, must be equally considered to those more typically focused on by app developers, namely the user interface and keeping the user engaged. Lastly, there is a need for rigorous evaluation and refinement of these apps using high-quality feasibility testing and multicenter randomized controlled trials.

### Conclusions

The overall conclusions advanced in this review are that despite the high accessibility of mobile apps for weight management, the quality of their content and functionality remains poor. Efforts to address these problems must involve health care experts during the app development process, a more comprehensive grounding in evidence-based practice, improved personalization and tailored feedback, and evaluation and refinement through scientific trials. The potential for mobile apps to improve health outcomes in the management of chronic diseases presents a real opportunity for widespread, cost-effective delivery of health care. Future research is urgently needed to develop comprehensive, evidence-based, and clinically-informed weight management mobile tools toward these aims.

## Abbreviations

BMI: body mass index
OS: operating systems
